# The Role of Endothelin System in Renal Structure and Function during the Postnatal Development of the Rat Kidney

**DOI:** 10.1371/journal.pone.0148866

**Published:** 2016-02-12

**Authors:** María F. Albertoni Borghese, María C. Ortiz, Sabrina Balonga, Rocío Moreira Szokalo, Mónica P. Majowicz

**Affiliations:** Cátedra de Biología Celular y Molecular, Departamento de Ciencias Biológicas, Facultad de Farmacia y Bioquímica, Universidad de Buenos Aires, Buenos Aires, Argentina; Universidad de La Laguna, SPAIN

## Abstract

Renal development in rodents, unlike in humans, continues during early postnatal period. We aimed to evaluate whether the pharmacological inhibition of Endothelin system during this period affects renal development, both at structural and functional level in male and female rats. Newborn rats were treated orally from postnatal day 1 to 20 with vehicle or bosentan (Actelion, 20 mg/kg/day), a dual endothelin receptor antagonist (ERA). The animals were divided in 4 groups: control males, control females, ERA males and ERA females. At day 21, we evaluated renal function, determined the glomerular number by a maceration method and by morphometric analysis and evaluated possible structural renal alterations by three methods: 〈alpha〉-Smooth muscle actin (α-SMA) immunohistochemistry, Masson's trichrome and Sirius red staining. The pharmacological inhibition of Endothelin system with a dual ERA during the early postnatal period of the rat did not leads to renal damage in the kidneys of male and female rats. However, ERA administration decreased the number of glomeruli, the juxtamedullary filtration surface area and the glomerular filtration rate and increased the proteinuria. These effects could predispose to hypertension or renal diseases in the adulthood. On the other hand, these effects were more pronounced in male rats, suggesting that there are sex differences that could be greater later in life. These results provide evidence that Endothelin has an important role in rat renal postnatal development. However these results do not imply that the same could happen in humans, since human renal development is complete at birth.

## Introduction

Endothelin (ET) system is represented by three structurally similar endogenous 21-aminoacid peptides named ET-1, ET-2 and ET-3, that activate two G-protein-coupled receptors ET_A_ and ET_B_ and two activating proteases [1]. Each isoform is encoded by a separate gene and both their synthesis and secretion are highly regulated at the transcriptional level by hormonal and environmental factors [2].

The different ET isoforms and the other components of this system are widely expressed in the body in most tissues, being ET-1 the predominant isoform [3,4].

At the renal level ET modulates blood flow, sodium and water excretion and acid-base balance [5]. In addition, it is well known that ET stimulates the transcription of different genes promoting mesangial glomerular and vascular smooth muscle cells proliferation, in addition to its ability to promote the synthesis of extracellular matrix [6].

The importance of ET system during development was demonstrated in KO mice, since the inactivation of any component of ET system leads to a lethal phenotype [7–11].

Despite definitive demonstrations of the crucial role of the ET system in early embryonic development, little is known about the importance of the ET system during the perinatal period, including the late embryonic and early neonatal stages [12]. In addition, the molecular basis of maturation during the early postnatal period remains very poorly understood and underinvestigated [13].

Gene expression of ET_A_ and ET_B_ receptors increases during the later stages of embryonic development in lung, heart, liver, kidney, and skin and reaches a maximum on the first one or two days after birth, suggesting that the ET system may be involved in the emergence and maintenance of vital functions after birth in these organs [14].

Increased ET receptor density in early postnatal life suggests an influence of ET-1 on immature kidney circulation and/or kidney growth [15]. Yoo et al showed that the inhibition of endogenous ETs by the administration of an antagonist of ET_A_ receptors to neonate rats impairs renal growth, with decreased cellular proliferation and increased apoptosis in the kidneys [16].

The blockade of angiotensin II (AII) effects during renal postnatal development in rats leads to a reduction in nephron number, a decreased ability to eliminate a volume overload, a decreased ability to concentrate the urine after dehydration and an impairment of both cortical and medullar function, being these effects sex and age dependent [17–19]. AII is closely related with ET-1 since many renal effects of AII are mediated by ET-1 [20,21].

Renal development in rodents continues during early postnatal period [22,23], in contrast with humans that complete their renal development in uterus.

Based on these antecedents, the aim of this study was to evaluate whether the pharmacological inhibition of ET system during the early postnatal period affects renal development, both at structural and functional level. In addition, we intended to analyze sex differences. To achieve this purpose, male and female newborn rats were treated with Bosentan, a dual endothelin receptor antagonist (ERA), during the early postnatal period.

## Materials and Methods

### Animals and treatments

Sprague Dawley (SD) rats were purchased from the School of Veterinary Sciences from the University of Buenos Aires. Protocols were designed according to the National Institutes of Health Guide for the Care and Use of Laboratory Animals, the American Physiological Society “Guiding Principles in the Care and Use of Animals” and with the 6344/96 regulation of Argentinean National Drug Food and Medical Technology Administration (ANMAT) and were approved by the Institutional Committee for Use and Care of Laboratory Animals from the School of Pharmacy and Biochemistry. All rats were housed in rooms with controlled temperature (24°C) and 12 h. dark-light cycle. Food and water were supplied ad libitum. Female SD rats (250 g body weight) were mated by exposure to a fertile SD male during 1 week.

After birth, litter size was fixed in 10±1. Litters with less than 8 pups were excluded. Newborn rats were treated daily from postnatal day 1 to postnatal day 20 with vehicle (distilled water) or with Bosentan (Actelion, 20 mg/kg/day), a dual ERA, which was administered via oral with a micropipette. Blockade of ET receptors was performed during the first 20 days of life, comprising all the lactation period, because in rats growth and maturation of the kidney also continue after the completion of nephrogenesis and it has been considered that nephrons reach terminal differentiation at the time of weaning [24,25].

The animals were divided in four groups: control males (Cm), control females (Cf), ERA males (ERAm) and ERA females (ERAf). The total number of animals per group and sex was: Cm: n = 18; ERAm: n = 20; Cf: n = 18; ERAf: n = 17.

The weight of the animals was registered daily with an electronic scale (Precision; model: TH-1000 series).

After weaning (day 21), the animals were placed in metabolic cages to obtain 24 h. urine samples. Then, the animals were anesthetized with urethane (1g/Kg) via i.p and blood samples were obtained by cardiac puncture, both kidneys were immediately removed and weighed. The right kidney of each animal was used to perform the count of glomerular number meanwhile the left kidney of each animal was used to perform histological evaluation, morphometric analysis and immunohistochemistry. Kidney weight was measured and expressed per 100g of body weight. Femur length was measured using a caliber.

### Determinations in the 24-hour metabolic cage studies

Twenty-four hour urine samples were collected using metabolic cages. Urine volume was measured gravimetrically. Urine samples were analyzed for total protein using a kit provided by Wiener (Proti U/LCR; Wiener Lab., Rosario, Argentina). Urinary sodium and potassium concentrations were evaluated using an ion analyzer (Tecnolab; Mod. T-412). Kinetic determinations of serum and urinary creatinine concentrations were evaluated using a kit provided by Wiener (Wiener Lab., Rosario, Argentina). It is known that creatinine clearance can overestimate glomerular filtration rate (GFR) in rodents on account of tubular secretion of creatinine. However, other methods such as inulin clearance are not simple and also have practical limitations [26], which are magnified when applied to 21-day-old rats.

### Count of glomerular number

A modification of the maceration method described by Damadian et al was used to count the glomerular number [27,28]. Briefly, the kidneys were decapsulated, cut into small pieces and incubated in 1% NH_4_Cl at room temperature, followed by incubation in 30 mL of 50% HCl for 90 min at 37°C with gently agitation. After slow-speed centrifugation, the pellet containing the glomeruli was suspended in 25 mL of distilled water. Twenty 20 μL aliquots were pipetted onto slides and all glomeruli were counted at 400x magnification. Although some experts favor stereology over maceration, the latter technique was chosen because it is simple and rapid and allows the detection of differences between groups [29]. The number of total glomeruli per rat was calculated as follows: [(N° of glomeruli per right kidney/ right kidney weight) x weight of both kidneys].

### Histological evaluation and morphometric analysis

The left kidneys were decapsulated and cut longitudinally, fixed in phosphate buffered 10% formaldehyde, pH 7.4, embedded in paraffin wax and cut to a thickness of 5μm. Renal tissue sections were stained with hematoxylin and eosin (H-E). To count the glomerular number, ten consecutive cortical and juxtamedullary areas from two renal sections per animal (five animals per group) were examined. The number of glomeruli was measured at 1 × 100 magnification and expressed per mm^2^.

To determine glomerular areas we analyzed at least ten cortical and ten juxtamedullary glomeruli from five animals of each group. Both total glomerular area and glomerular capillary area were expressed in μm^2^ and the ratio capillary glomerular area/total glomerular area (CGA/TGA) of cortical and juxtamedullary nephrons were determined at 1 × 400 magnification.

The filtration surface area of renal juxtamedullary and cortical tissue was calculated as the product of mean glomerular capillary area by the number of glomeruli per mm^2^.

To determine the presence of early fibrosis in the renal cortex, kidney sections were subjected to 〈alpha〉-Smooth muscle actin (α-SMA) immunohistochemistry, Masson's trichrome and Sirius red staining. At least ten cortical and ten juxtamedullary fields from five animals of each group were analyzed.

### Immunohistochemistry

To perform immunohistochemistry a primary mouse monoclonal antibody to α-SMA was used (Biogenex, Canyon Road San Ramon, CA 94583, USA). Then, the kidney sections were incubated with a secondary biotinylated donkey anti mouse (Jackson ImmunoResearch, West Grove, PA, USA) followed by the streptavidin-biotin-peroxidase reaction (Dako Cytomation, Glostrup, Denmark) and visualized by exposure to dioaminobenzidine (DAB)-H_2_O_2_. Negative controls were performed by omitting the primary antibody and endogenous peroxidase activity was quenched with hydrogen peroxide to prevent unspecific staining. Tissue sections were counterstained with hematoxylin.

Expression of α-SMA in the renal cortex was scored as follows: 0: normal staining confined only to smooth muscle cells of blood vessels; 1: (mild) additional weak staining in the peritubular interstitium, glomeruli, and periglomerular structures; 2: (moderate) moderate segmental or focal staining of peritubular interstitium, periglomerular structures, and a small minority of glomeruli; 3: (severe) strong staining, 25% of the cortical area, in the majority of glomerular cells and tubular and peritubular structures; and 4: (very severe) strong staining, >25% of the cortical area, in the majority of glomerular cells and tubular and peritubular structures. A score was assigned to each section, mainly reflecting the changes in the extent rather than the intensity of staining [30].

### Sirius red staining

Collagen accumulation was examined in the renal sections with the collagen-specific stain picrosirius red (Sirius Red 3 in a saturated aqueous solution of picric acid and fast green as a counterstain). Sirius Red staining is a method for collagen determination, enabling quantitative morphometric measurements to be performed in locally defined tissue areas [31]. Staining was scored as 0 (normal and slight staining surrounding the tubular, glomerular, and vascular structures), 1 (weak staining that doubles the normal label surrounding the tubular, glomerular, and vascular structures), 2 (moderate staining in the peritubular interstitium and inside the glomeruli), 3 (strong staining that replaces the glomerular and tubular structures, compromising <25% of the cortical area), or 4 (strong staining that replaces the glomerular and tubular structures, compromising>25% of the cortical area).

### Masson´s trichrome staining

Masson’s trichrome staining was carried out and the proportion of blue-stained fibrotic area in the cortex of each section was graded semiquantitatively (0: ≤5%, 1: 5% to 25%, 2: 25% to 50%, 3: 50% to 75%, 4: ≥75%). These examinations were performed by two investigators without knowledge of the origin of the slides, and the mean values were calculated [32].

### Image capture and analysis

Images from histological and immunohistochemical sections were captured using a Nikon Alphaphot-2 YS2 light microscope (Nikon Instrument Group, Melville, NY), coupled to a Sony color video camera digital (Model N° SSC-DC50A). All determinations were performed blindly and under similar light, gain and offset conditions by the same researcher. Image-Pro Plus 5.1 Ink software (Media Cybernetics, LP, Silver Spring, MD) was used to evaluate glomerular areas and fibrosis.

### Statistical analysis

Data are presented as the mean ± standard error of the mean. Data were analyzed using two-way ANOVA where one factor was the treatment (control or ERA) and the other was the sex of the animals (male or female rats). The main effect of each factor was tested as well as the interaction within both factors. Bonferroni´s post-test was used for multiple comparisons. When the interaction was found to be statistically significant, the main effect of each factor was not informed (as each factor is influenced by the other) and simple main effects were informed separately. Data were analyzed using Graph Pad Prism version 5.0 for Windows, Graph Pad Software (San Diego, CA, USA). The null hypothesis was rejected when p<0.05.

## Results

### Effect of ERA administration on growth parameters

The body weight of ERA-treated rats (male and female) decreased at the end of treatment when compared with control groups (male and female respectively). However, there were no differences between groups in femur length. We did not find differences in kidney weight, expressed per 100g of body weight. These results are shown in Table 1.

**Table 1:** Growth parameters at day 21 of life in control and ERA-treated male and female rats.

	Cm	ERAm	Cf	ERAf
Body weight (g)	44.81±0.74 (n = 18)	40.69±0.96** (n = 20)	43.79±1.15 (n = 18)	39.68±0.92## (n = 17)
Renal weight (g/100g b.w.)	1.24 ± 0.04 (n = 18)	1.30 ± 0.04 (n = 18)	1.29 ± 0.03 (n = 16)	1.32±0.03 (n = 16)
Femur length (cm)	1.71±0.02 (n = 11)	1.70±0.02 (n = 11)	1.69±0.02 (n = 10)	1.69±0.02 (n = 10)

ERA: Endothelin receptor antagonist. Cm: control males; ERAm: ERA males; Cf: control females; ERAf: ERA females. Values are mean ± SEM. Data were analyzed using two way ANOVA followed by Bonferroni posttest. There was no significant interaction between the administration of ERA and sex. Treatment with ERA had a significant overall effect on final body weight. Sex had no significant effect on any of the parameters tested.

** p<0.01 vs Cm;

^##^ p<0.01 vs Cf.

### ERA administration during early postnatal life decreases nephron number and affects renal filtration surface area

**Fig 1:**
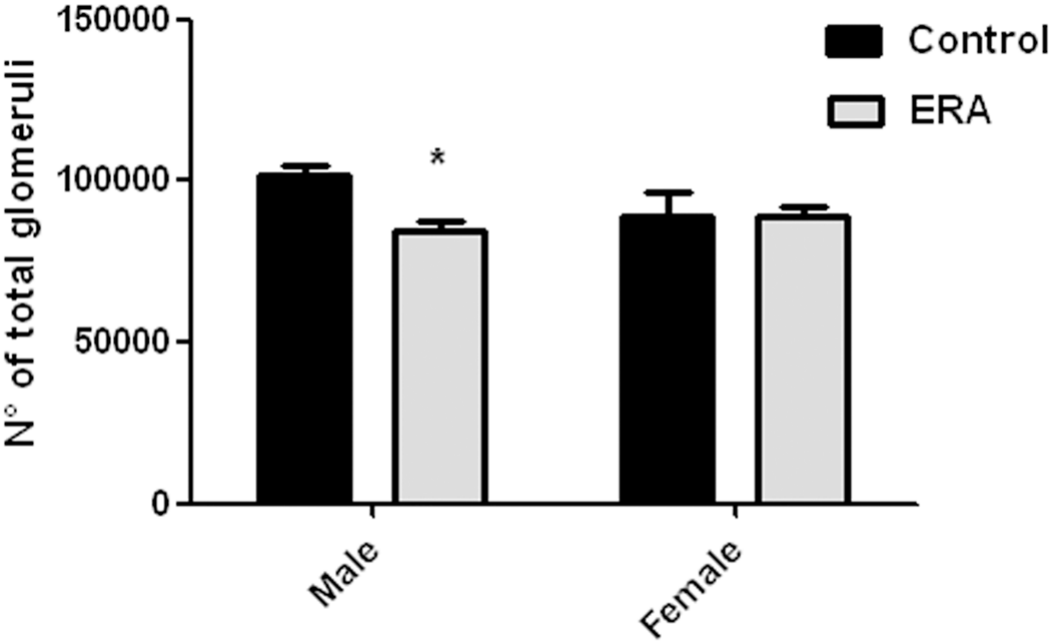
Number of total glomeruli. Number of total glomeruli obtained by the maceration method. ERA: Endothelin receptor antagonist. Values are mean ± SEM (n = 10). Data were analyzed using two way ANOVA followed by Bonferroni posttest. Two-way ANOVA showed a statistically significant interaction (p < 0.05) between the effects of ERA treatment and sex. *p<0.05 vs control males.

The number of total glomeruli determined by the maceration method significantly decreased in ERAm vs Cm (Fig 1).

In the cortical area, the treatment with the ERA decreased the CGA to the same extent in male and female rats. Female groups (control and ERA-treated) showed a lower CGA than their respective male groups.

There were no significant changes in the other morphometric parameters evaluated in the cortical area; although there was a tendency to decrease renal filtration surface area and CGA/TGA % in ERAm-treated rats.

The main changes in the morphometric analysis were observed in the juxtamedullary area (JA). The morphometric evaluation showed that the number of glomeruli/mm^2^ decreased significantly in the JA of both ERAm and ERAf compared with their respective controls. In addition, the juxtamedullary renal filtration surface area significantly decreased in both ERAm and ERAf. The juxtamedullary capilar glomerular area (CGA) significantly decreased in both ERAm and ERAf. The effect of ERA treatment on juxtamedullary capilar glomerular area/total glomerular area (CGA/TGA) ratio was different in females than in males. There was a significant decrease in the CGA/TGA ratio of the juxtamedullary nephrons in ERAf, whereas there was only a tendency to decrease this parameter in ERAm.

When we determined the number of glomeruli/mm^2^ without differenciating between CA and JA, we found significant differences in ERAm when compared with Cm, a result concordant with that obtained by the maceration method. hese results are shown in Table 2.

**Table 2:** Morphometric parameters in juxtamedullary and cortical renal tissue.

	Cm	ERAm	Cf	ERAf
**CORTICAL AREA**				
Total glomerular area (TGA, μ^2^) [CA]	3007±68	2978±64	2899±132	2882±89
Capilar glomerular area (CGA, μ^2^) [CA]	2478±63	2333±53**	2274±115**	2139±64##&&
CGA/TGA % [CA]	80.7±0.6	78.4±0.7	75.5±1.7	76.3±3.3
Renal filtration surface area (μm^2^) [CA]	66117±2582	58444±3146	63740±3019	62288±4143
N° of glom/mm^2^ [CA]	26.5±1.2	25.1±1.4	28.2±0.9	29.3±2.2
**JUXTAMEDULLARY AREA**				
Total glomerular area (TGA,μm^2^) [JA]	4694±150	4372±119	4355±200	4478±185
Capilar glomerular area (CGA, μm^2^) [JA]	3774±129	3478±100*	3626±197	3304±141#
CGA/TGA % [JA]	80.4±0.8	79.7±0.8	83.3±1.6	76.2±0.9###
Renal filtration surface area (μm^2^) [JA]	47872±2953	35011±2840**	47077±2732	37765±2488##
N° of glom/mm^2^ [JA]	12.9±0.8	10.2±0.8**	13.4±0.9	11.2±0.9##
**TOTAL CORTEX AREA (CA+JA)**				
N° of glom/mm^2^	19.3±2.3	15.0±1.0*	19.1±0.9	18.9±2.2

ERA: Endothelin receptor antagonist. Cm: control males; ERAm: ERA-treated males; Cf: control females; ERAf: ERA-treated females. Values are mean ± SEM. CA: cortical area; JA: juxtamedullary area. Data were analyzed using two way ANOVA followed by Bonferroni posttest. n = 5 animals/group and at least 10 fields/animal were analyzed.

*p<0,05 vs Cm;

** p<0,01 vs Cm;

^#^ p<0.05;

^##^ p<0,01 vs Cf;

^###^ p<0,001 vs Cf;

^&&^ p<0.01 vs ERAm.

Two-way ANOVA showed a statistically significant interaction (p < 0.05) between the effects of ERA treatment and sex on CGA /TGA % (JA) and in number of glom/mm^2^ (CA + JA). There was no interaction between the effects of ERA treatment and sex on the other parameters. ERA treatment had a significant overall effect on CGA (CA) (p<0.01), CGA (JA) (p<0.05), N° of glom/mm^2^ (JA) (p<0.01) and renal filtration surface area (JA) (p<0.01). Sex had a significant overall effect (p<0,05) on CGA (CA).

### Effect of ERA administration on renal functional parameters

**Fig 2:**
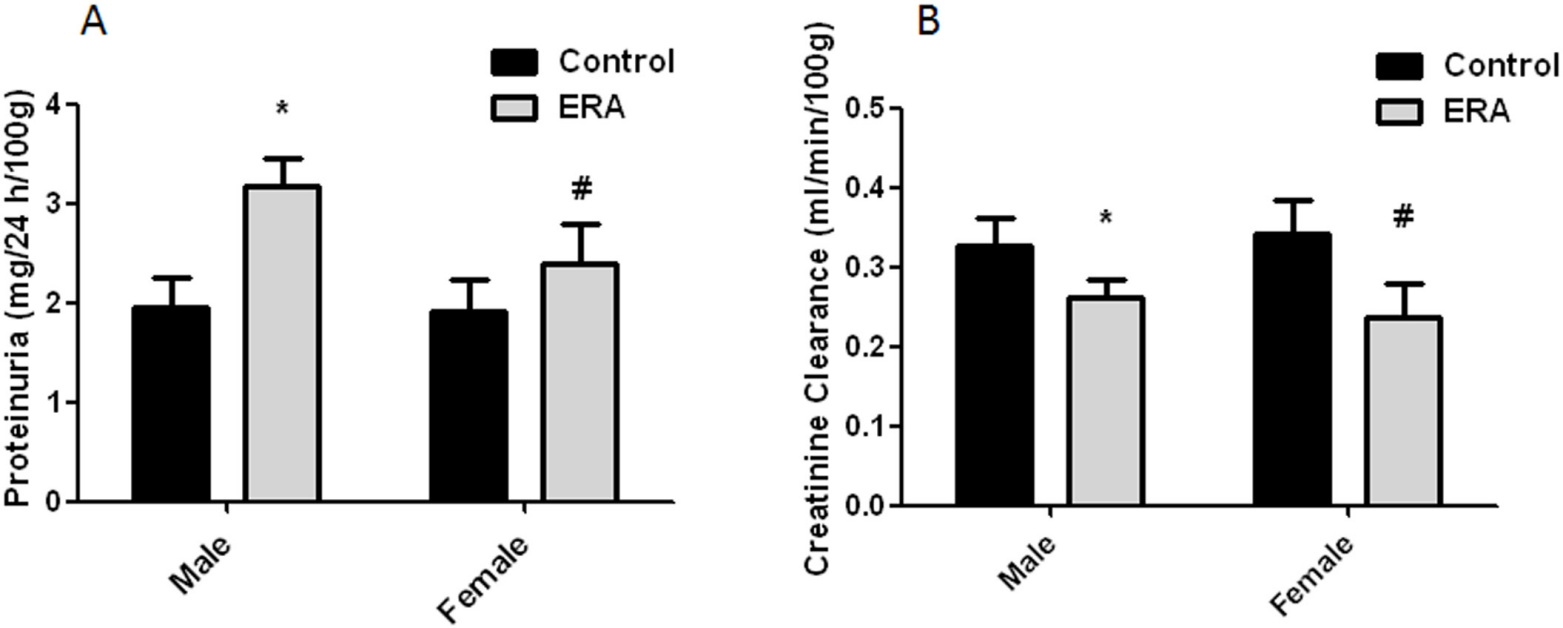
A: Proteinuria (mg/24 h/100g). B: Creatinine Clearance (ml/min/100g). ERA: Endothelin receptor antagonist. Values are mean ± SEM. Data were analyzed using two way ANOVA followed by Bonferroni posttest.

There was a significant increase in proteinuria in both male and female ERA-treated rats versus their respective control groups, being this increase higher in male than in female animals (Fig 2A). GFR, estimated as the clearance of creatinine, significantly decreased in both male and female ERA-treated rats (Fig 2B). This decrease could be explained by the diminished renal filtration surface, which in turn could be a consequence of the smaller capilar glomerular area and/or the decreased number of glomeruli. There were no significant changes in the other renal functional parameters evaluated, although there was a tendency to increase diuresis. These results are shown in Table 3.

**Table 3:** Renal functional parameters.

	Cm	ERAm	Cf	ERAf
Diuresis (ml/24hs/100g)	3.79±0.48 (n = 12)	4.52±0.50 (n = 14)	3.96±0.50 (n = 12)	4.63±0.67 (n = 12)
Natriuresis (mEq/24hs/100g)	0.89±0.20 (n = 12)	1.10±0.17 (n = 14)	1.47±0.31 (n = 12)	1.20±0.23 (n = 12)
Kaliuresis (mEq/24hs/100g)	1.12±0.18 (n = 12)	1.31±0.11 (n = 14)	1.48±0.20 (n = 12)	1.30±0.17 (n = 12)

ERA: Endothelin receptor antagonist. Cm: control males; ERAm: ERA-treated males; Cf: control females; ERAf: ERA-treated females. Values are mean ± SEM. Data were analyzed using two way ANOVA followed by Bonferroni posttest. ERA treatment and sex had no significant effect on any of the parameters tested.

There was no interaction between the effects of ERA treatment and sex. ERA treatment had a significant overall effect on proteinuria and creatinine clearance (*p<0.05 vs control males; # p<0.05 vs control females).

### ERA administration during early postnatal life does not lead to early renal morphologic alterations in the kidneys of male and female rats

**Fig 3:**
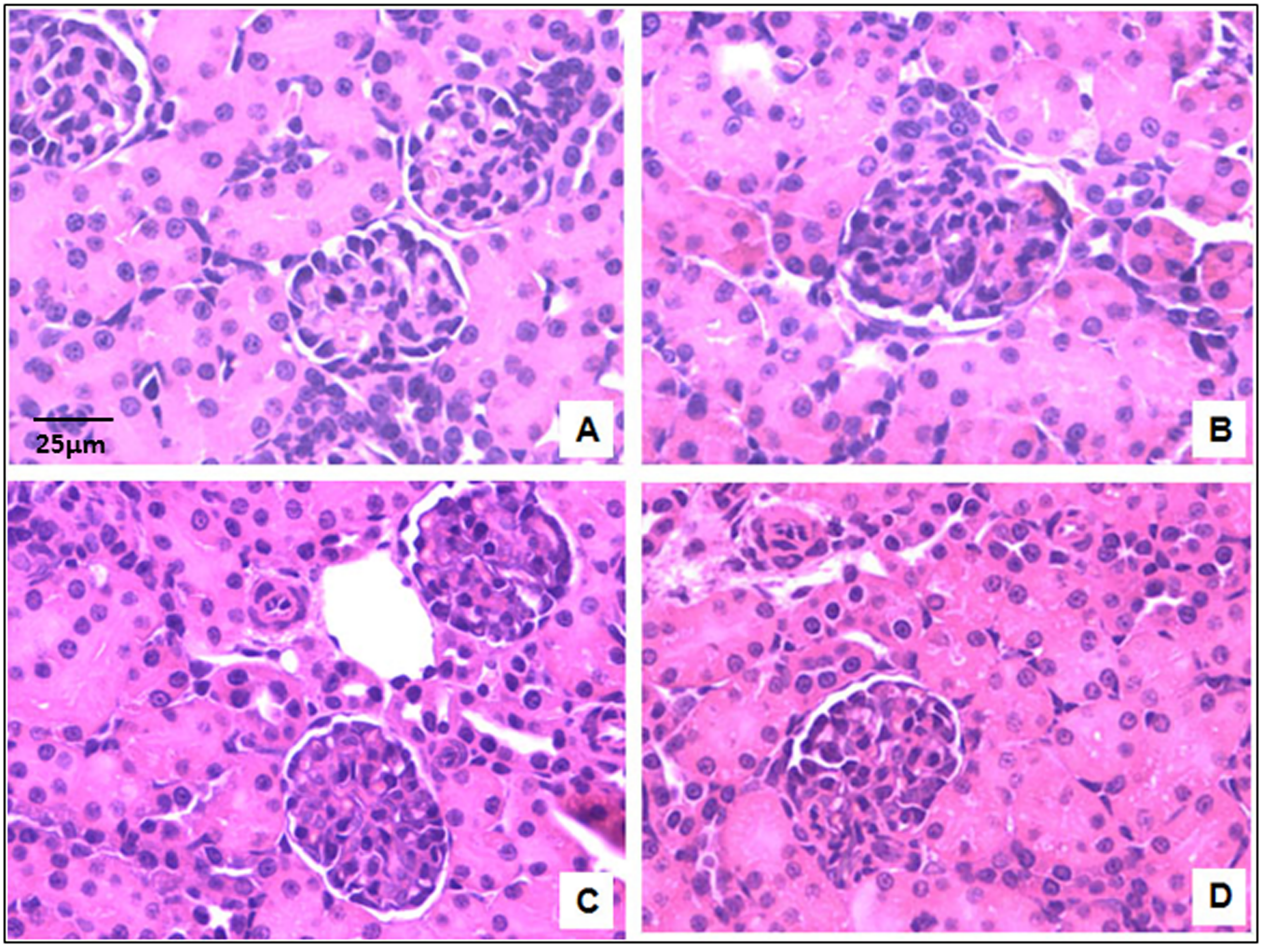
Histological structure of the renal cortex. Representative images of Hematoxylin-eosin staining of the renal cortex. A = control males; B = ERA-treated males; C = control females; D = ERA-treated females. ERA: Endothelin receptor antagonist. Total magnification: 400x.

**Fig 4:**
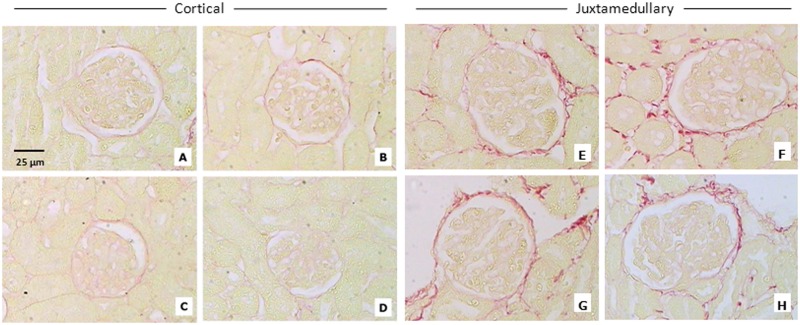
Sirius red staining of the renal cortex. Representative images of Sirius red staining of the renal cortex; CA: cortical area; JA: juxtamedullary area. A = control males (CA); B = ERA-treated males (CA); C = control females (CA); D = ERA-treated females (CA); E = control males (JA); F = ERA-treated males (JA); G = control females (JA); H = ERA-treated females (JA). ERA: Endothelin receptor antagonist. Total magnification: 400x.

**Fig 5:**
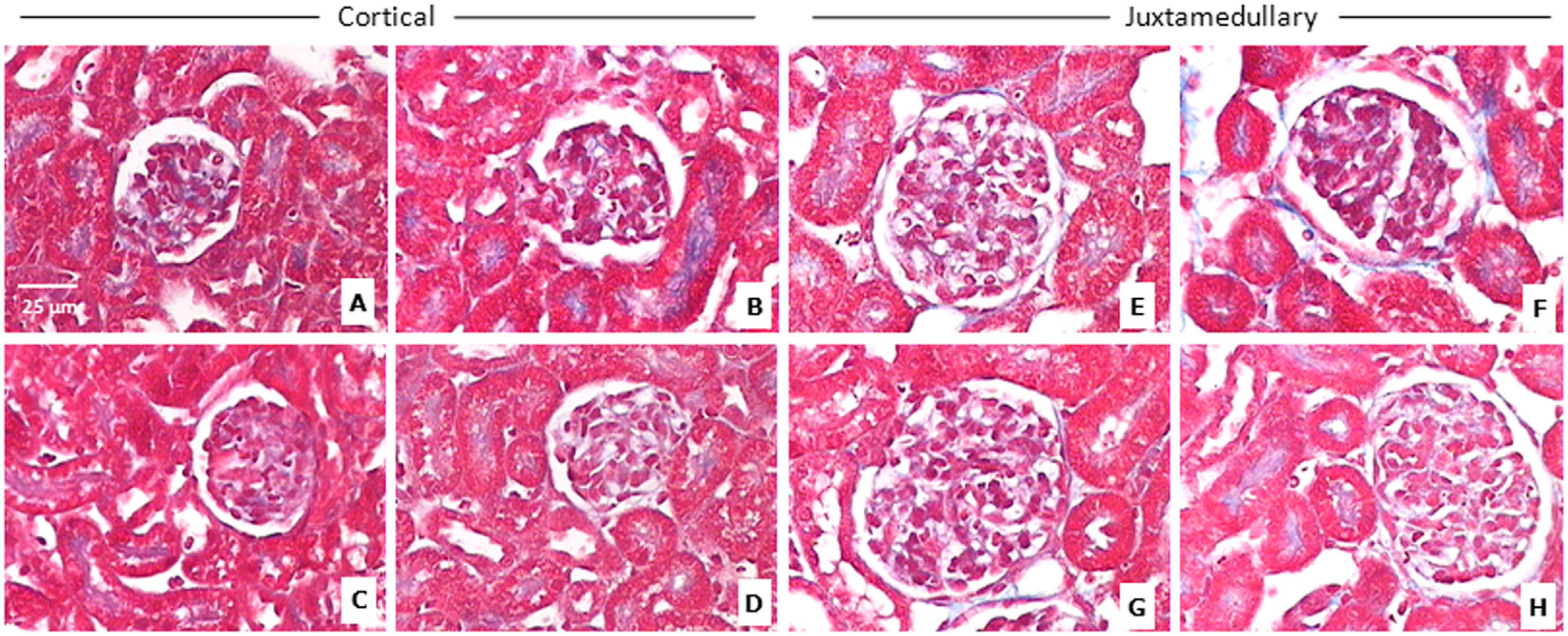
Masson´s trichrome staining of the renal cortex. Representative images of Masson´s trichrome staining of the renal cortex; CA: cortical area; JA: juxtamedullary area. A = control males (CA); B = ERA-treated males (CA); C = control females (CA); D = ERA-treated females (CA); E = control males (JA); F = ERA-treated males (JA); G = control females (JA); H = ERA-treated females (JA). ERA: Endothelin receptor antagonist. Total magnification: 400x.

**Fig 6:**
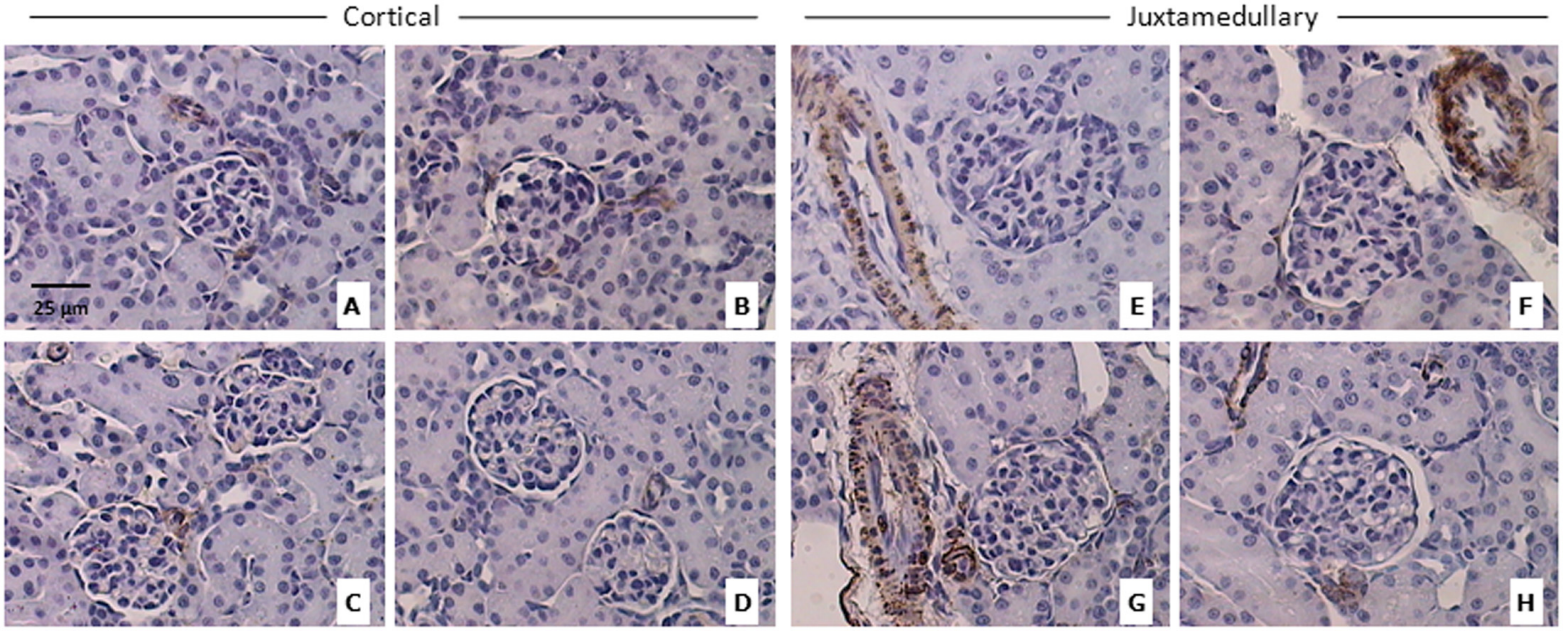
α-SMA immunohistochemistry of the renal cortex. Representative images of α-SMA immunohistochemistry of the renal cortex; CA: cortical area; JA: juxtamedullary area. A = control males (CA); B = ERA-treated males (CA); C = control females (CA); D = ERA-treated females (CA); E = control males (JA); F = ERA-treated males (JA); G = control females (JA); H = ERA-treated females (JA). ERA: Endothelin receptor antagonist. Total magnification: 400x.

The histological structure of rat kidneys in the H-E sections seemed to be unaffected (Fig 3). The score for both Masson´s trichrome and Sirius red staining was <1 for all the groups; it means a normal and slight staining surrounding tubular, glomerular and vascular structures (Table 4). The score for α-SMA was 0 for all the groups, being the staining normal and confined only to smooth muscle cells of blood vessels. Representative images of the three techniques can be observed in Figs 4–6.

**Table 4:** Renal histochemical evaluation by Sirius red and Masson´s thrichrome.

	Cm	ERAm	Cf	ERAf
Sirius red score (CA)	0.21±0.08	0.27±0.08	0.30±0.10	0.25±0.08
Sirius red score (JA)	0.35±0.09	0.65±0.09	0.44±0.12	0.40±0.11
Masson`s trichrome score (CA)	0.53±0.08	0.38±0.06	0.50±0.10	0.47±0.11
Masson`s trichrome score (JA)	0.44±0.08	0.32±0.07	0.71±0.08	0.53±0.10

ERA: Endothelin receptor antagonist. Cm: control males; ERAm: ERA-treated males; Cf: control females; ERAf: ERA-treated females; CA: cortical area; JA: juxtamedullary area. Values are mean ± SEM. Data were analyzed using two way ANOVA followed by Bonferroni posttest. n = 5 animals/group and at least 10 fields/animal were analyzed.

## Discussion

It is well known that ET plays a central role in renal sodium and water balance, arterial blood pressure regulation and the development and maintenance of kidney disease in adult animals [33]. However, there are only a few studies about the participation of ET system during the postnatal renal development. The importance of this study lies in the fact that the stimuli or impact that an organ receives during its perinatal development (in this case the administration of a dual ERA) may impact in its function during the adulthood.

The present study demonstrates that the pharmacological blockade of ET during the early postnatal period impairs body weight in both male and female rats. However, this result cannot be explained by a defect in the growth of the animals due to the absence of changes in femur length between the different experimental groups. In addition, there were no significant differences in renal weight between control and ERA-treated rats. The differences seen in body weights could be reflecting a greater water loss in ERA-treated rats than in control rats. Although the diuresis did not differ significantly between control and ERA-treated rats, there was a tendency to increase this parameter; a possible explanation may be that the increase in water loss could be due to decreased water reabsorption at tubular level. Alternatively, it may be that the differences in body weights of ERA-treated rats reflect a diminished food intake.

No signs of structural renal damage were observed at the end of the treatment in the ERA-treated rats, evaluated by both histochemical and immunohistochemical methods. However, it would be interesting to evaluate if there are signs of fibrosis in these animals at a later stage of their lives. Sometimes early morphological changes are not evident, but changes at the molecular level are present and manifest later in life.

The histological structure of rat kidneys in the H-E sections seemed to be unaffected. However, the morphometric analysis showed a decrease in glomerular number in the juxtamedullary area of the renal cortex for both ERAm and ERAf. The decrease in glomerular number seen in the morphometric analysis in ERAm was coincident with the decrease in the glomerular number observed by the maceration method. However, the maceration method did not show a decrease in glomerular number in ERAf. A decrease in nephron number has been found to be associated with susceptibility to develop diseases like hypertension and chronic renal failure [34–37]. This reduced nephron number seen in ERA-treated rats may be a consequence of decreased cellular proliferation or increased apoptosis in the renal cortex of the animals. Tight regulation of apoptosis is required in normal renal morphogenesis, being regulated by genetic, epigenetic and environmental factors, thus dysfunctions in apoptosis can manifest as developmental abnormalities [38]. In fact, Yoo et al have shown that inhibition of endogenous endothelins during the postnatal period impairs renal growth, in which decreased cellular proliferation, increased apoptosis and decreased expressions of renal Bcl-X(L) and Bax are possibly implicated [39]. However, they used a selective ET_A_ receptor antagonist only from postnatal day 1 to 7. It has been shown that ET-1 receptor activation results in the stimulation of several signaling pathways including MAPKs/ERK and PI3-K [40]. Activation of the ETA or the ETB receptor results in phosphorylation of ERK, which is an important regulator for cellular proliferation, migration, differentiation and vascular smooth muscle constriction [41]. In addition, ET-1 induces activation of RAS in rats and human renal mesangial cells, which is dependent upon the formation of the Shc/Grb2/Sos1 signalling complex and resulted in ERK activation [42]. On the other hand, PI3K signaling is involved in growth and survival, inducing apoptosis inhibition. In mesangial cells, ET-1 receptor activation has been shown to stimulate PI3-K phosphorylation through Ras and to increase the catalytic activity of PI3-K [43,44].

So, it is probable that the blockade of both ET receptors can lead to the principal changes reported by inhibition of the MAPKs signaling and/or inhibition of PI3-K signaling. The decrease in the number of juxtamedullary glomeruli and consequently in renal filtration surface area observed in ERam and ERAf rats could be due to decreased cellular proliferation and increased apoptosis. These cellular events could be mediated by endothelin dependent signaling activating pathways that have implications in regulating proliferation, survival and apoptosis.

The reduced nephron number that we observed was accompanied with a reduction of the renal filtration surface area at juxtamedullary level for both ERAm and ERAf. There were no significant changes in the renal filtration surface at cortical level although there was a tendency to decrease those values, especially in ERAm.

As can be seen in Table 2, both total and glomerular capilar areas were larger in juxtamedullary than in cortical zone. It is known that glomeruli that will be located in the juxtamedullary region develop first and are larger than superficial glomeruli at birth and during early postnatal life [45,46]. Another interesting finding is that in ERA-treated rats the proteinuria was significantly higher than in control rats. In addition, the proteinuria in ERAm was higher than in ERAf. The molecular mechanisms that lead to proteinuria are poorly understood [47], but bearing in mind that ET-1, ET_A_ and ET_B_ are expressed in both podocytes and glomerular endothelial cells [48], this increase in proteinuria observed in ERA-treated rats suggests that ET regulates the composition and/or the function of the glomerular filtration barrier during postnatal development. There is recent evidence that TGF-β1 plays crucial roles in podocyte differentiation, glomerulogenesis, and nephrogenesis during kidney development and podocyte injury responses [49]. Bearing in mind the interaction of ET-1 and TGF-β in diverse tissues and organs, including the kidney, it is tempting to speculate that ET and TGFβ interact during renal postnatal development acting on the glomerular filtration barrier formation.

The differences seen in neonatal male and female rats (hormone-independent) can be explained by the presence of epigenetic mechanisms that affect distinctively males and females. Epigenetic mechanisms that affect genes include insertion of histone variants, post-translational modifications of histones, expression of non-coding RNAs (ncRNAs) and methylation of DNA. These epigenetic effectors alter both the availability of genes for transcription and the rates of transcription. Two epigenetic mechanisms known to regulate the genes of the ET pathway are DNA methylation and histone modification. Most of the evidence available on epigenetic regulation of the ET pathway focuses on the EDN1 (gene that codifies ET-1) and EDNRB (gene that codifies ET_B_ receptor) [50].

The pharmacological inhibition of ET system with a dual ERA during the early postnatal period of the rat decreases the number of glomeruli, the juxtamedullary filtration surface area, the glomerular filtration rate and increases the proteinuria. These effects could predispose to hypertension or renal diseases in the adulthood. On the other hand, these effects were more pronounced in male rats, suggesting that there are sex differences that could be greater later in life when sex hormones play a role. It is known that sex hormones affect Endothelin plasma levels, which are increased by testosterone and decreased by oestradiol [51]. In addition, sex steroids influence almost every component of the ET system [52–55]. Renal ET receptors function somewhat differently between males and females. ET_A_ receptor activation leads to unfavorable effects in male kidneys, including renal medullary vasoconstriction and renal injury. In contrast, females are relatively protected against high blood pressure and kidney damage by virtue of increased ET_B_ receptor function and perhaps reduced ET_A_-dependent haemodynamic effects [56].

These results provide evidence that ET has an important role in rat renal postnatal development. However, these results do not imply that the same could happen in humans, since human renal development is complete at birth. Our study could be clinically compared with the 3rd trimester of human gestation or the premature human kidney and actually highlights the importance of this type of studies to develop therapeutic approaches during perinatal life.
